# Postpartum Reverse Takotsubo Cardiomyopathy After Cesarean Delivery in the Setting of War‐Related Stress: A Case Report and Literature Review

**DOI:** 10.1002/ccr3.73246

**Published:** 2026-08-20

**Authors:** Ali Khalil, Salwa Abouljoud, George Hatoum, Nathalie Chamseddine, Omar Alameddine, Ghina Ghazeeri

**Affiliations:** ^1^ Department of Obstetrics and Gynecology, Faculty of Medicine American University of Beirut Medical Center Beirut Lebanon

**Keywords:** emotional stress, peripartum cardiomyopathy, postpartum, reverse Takotsubo, stress‐induced cardiomyopathy, Takotsubo cardiomyopathy

## Abstract

Reverse Takotsubo syndrome should be considered in postpartum patients presenting with acute heart failure, particularly under extreme emotional stress. Early recognition and differentiation from peripartum cardiomyopathy are essential, given its rapid reversibility and favorable prognosis.

## 1 Introduction

Takotsubo cardiomyopathy, also known as stress‐induced cardiomyopathy, is an acute cardiac disease characterized by transient left ventricular systolic dysfunction in the absence of obstructive coronary artery disease. It may mimic acute coronary syndrome, presenting with chest pain, electrocardiographic changes, and elevated cardiac biomarkers [1, 2].

Although traditionally considered benign and reversible, recent evidence suggests that Takotsubo syndrome may be associated with significant short‐term complications and long‐term morbidity [3]. The pathophysiology remains multifactorial, involving coronary microvascular dysfunction, catecholamine excess, and dysregulation of the brain–heart axis in response to stress [4].

This condition predominantly affects postmenopausal women, while its occurrence during pregnancy and the postpartum period is rare. Limited data estimate an incidence of approximately 3 cases per 100,000 pregnancies [5]. In this setting, it presents a diagnostic challenge due to overlap with more common causes of postpartum heart failure, particularly peripartum cardiomyopathy.

Among its variants, the typical apical ballooning pattern is the most common [6, 7], while the reverse variant, characterized by basal hypokinesis and apical hyperkinesis, is less frequent and seems to occur more commonly in the younger population, including those in the peripartum period [8].

The peripartum period by itself constitutes a precipitating factor, owing to multiple potential triggers, such as the abrupt hormonal changes, surgical stress, and intense emotional stress [6].

We report a case of reverse Takotsubo syndrome at the American University of Beirut Medical Center (AUBMC), Beirut, Lebanon, that occurred in the immediate postpartum period during substantial emotional stress related to regional war. This case stresses the importance of early recognition of this rare variant in the differential diagnosis of acute cardiopulmonary presentation in the postpartum period.

## 2 Case History/Examination

A 32‐year‐old previously healthy primiparous woman with twin gestation conceived via in vitro fertilization underwent a primary cesarean section at 36 weeks of gestation due to preterm labor. The procedure was performed at another hospital under spinal anesthesia with no use of vasopressors without intraoperative complications.

The delivery occurred during active armed conflict in Lebanon, with nearby airstrikes prompting transfer to our tertiary care center.

Twelve hours postpartum, the patient developed mild dyspnea with oxygen desaturation to 89% on room air. She denied chest pain, palpitations, cough, fever, or chills. On physical examination, she was hemodynamically stable with no signs of volume overload.

## 3 Differential Diagnosis, Investigations, and Treatment

Initial laboratory investigations revealed elevated cardiac biomarkers, including a troponin level of 763 ng/L (reference range: 0–30 ng/L) and NT‐proBNP level of 1505 pg/mL (reference range < 116 pg/mL). Electrocardiography showed nonspecific changes.

Chest computed tomography demonstrated diffuse multilobar ground‐glass opacities with small bilateral pleural effusions and no evidence of pulmonary embolism. Although postpartum fluid shifts may have contributed to volume overload, these findings were most likely related to pulmonary edema and pulmonary congestion.

Transthoracic echocardiography revealed severely impaired left ventricular function with an ejection fraction (EF) of 25% and wall‐motion abnormalities characterized by basal hypokinesis and apical hyperkinesis, along with mild pericardial effusion.

Differential diagnoses included peripartum cardiomyopathy, pulmonary embolism, acute coronary syndrome, myocarditis, and other causes of postpartum pulmonary edema. Pulmonary embolism was excluded by imaging.

The diagnosis of Takotsubo cardiomyopathy was supported by the patient's clinical presentation, echocardiographic findings, and InterTAK Diagnostic Score. The patient was female and had a strong emotional trigger, with no ST‐segment depression on electrocardiography, corresponding to an InterTAK Diagnostic Score of 61 points. This score supports an intermediate‐to‐high probability of Takotsubo cardiomyopathy.

The patient was admitted to the cardiac unit and treated with intravenous furosemide, captopril, and bisoprolol. Further cardiac investigations, including cardiac MRI and left heart catheterization, were initially planned; however, they were deferred due to the patient's rapid clinical recovery.

Table 1 illustrates the exact timeline illustrating the sequence of clinical events.

**TABLE 1:** Timeline of the sequence of events from time of delivery until complete recovery.

Date/timing	Clinical event
01/10/2024	An uncomplicated primary cesarean section for twin gestation was performed under spinal anesthesia
01/10/2024 – Post‐delivery	Delivery occurred during active armed conflict in Lebanon, with nearby airstrikes prompting transfer to our tertiary care center
02/10/2024, 12 h postpartum	The patient developed mild dyspnea with oxygen desaturation to 89% on room air
02/10/2024—Initial workup	Laboratory testing showed elevated cardiac biomarkers, with troponin of 763 ng/L and NT‐proBNP of 1505 pg/mL. Electrocardiography showed nonspecific changes
	Chest CT demonstrated diffuse multilobar ground‐glass opacities with small bilateral pleural effusions and no evidence of pulmonary embolism
	Transthoracic echocardiography showed severely impaired left ventricular systolic function, with an EF of 25%, basal hypokinesis, apical hyperkinesis, and mild pericardial effusion
Within 24 h of treatment	Clinical improvement was noted
04/10/2024, 48 h after presentation	Repeat echocardiography showed improvement in left ventricular systolic function, with EF increasing to 45%
05/10/2024	The patient was discharged home hemodynamically and clinically stable
10/10/2024	First outpatient follow‐up visit showed continued clinical recovery
11/11/2024	Repeat outpatient echocardiography demonstrated complete recovery of left ventricular systolic function, with an EF of 60%–65%
1‐ and 6‐month follow‐up after discharge	The patient remained under close follow‐up. She was clinically stable and completely asymptomatic, with normal vital signs and no reported dyspnea, orthopnea, or chest pain

Breastfeeding was encouraged by all treating teams. The patient received counseling regarding the compatibility and safety of her medications during lactation; however, she ultimately decided not to breastfeed or breast‐pump.

## 4 Conclusion and Results

The patient's symptoms improved within 24 h. Repeat echocardiography at 48 h showed improvement in left ventricular function, with ejection fraction increasing to 45%.

She remained hemodynamically stable and was discharged with cardiology follow‐up. Subsequent outpatient evaluation demonstrated gradual clinical recovery, and she was maintained on angiotensin‐converting enzyme inhibitor therapy.

A repeat echocardiography performed 1 month later demonstrated complete recovery of left ventricular systolic function, with an EF of 60%–65%. The patient continued to remain under close follow‐up at 1 and 6 months after discharge. During follow‐up, she was clinically stable and completely asymptomatic, with normal vital signs and no reported dyspnea, orthopnea, or chest pain.

## 5 Discussion

Takotsubo cardiomyopathy is a reversible acute heart failure syndrome described by transient regional dysfunction in the left ventricular systolic function covering more than a solitary coronary territory. Sometimes referred to as “broken heart syndrome”, it is typically triggered by strong physical or emotional stress [1, 2]. Although the classic apical ballooning variant is the most common form, younger patients such as our patient are likely to present with atypical phenotypes, like reverse Takotsubo syndrome (rTTS), characterized by basal hypokinesis with apical hyperkinesis [3]. This variant has been described more frequently in peripartum and younger patients, hence its relevance in the postpartum diagnostic workups [4].

In our case, the acute postpartum onset along with the elevation in cardiac biomarkers, with echocardiographic pattern of basal hypokinesis with apical hyperkinesis, and the rapid improvement in left ventricular function within 48 h of onset strongly support the diagnosis of rTTS. Early reversibility is a key distinctive feature, as Takotsubo syndrome normally demonstrates quick recovery over days to weeks, compared to other causes of postpartum heart failure [2, 4].

Peripartum cardiomyopathy (PPCM) constitutes another important differential diagnosis in this case. PPCM is an idiopathic cardiomyopathy presenting mainly in the late third trimester to few months postpartum, with reduced left ventricular ejection fraction in the absence of another cause [6]. Clinically, Takotsubo cardiomyopathy and PPCM share overlapping presentations, including pulmonary congestion, dyspnea, elevated natriuretic peptides, and reduced ejection fraction [4, 6]. Nonetheless, numerous findings favor rTTS in this case, including the characteristic reverse pattern, the presence of regional rather than global wall‐motion abnormalities, and the rapid improvement in systolic function on repeat echocardiography. In contrast, in the absence of an identifiable cause, PPCM exhibits global systolic dysfunction, global wall‐motion abnormalities, and a less abrupt recovery trajectory [6].

A notable feature of this case is its temporal association with exposure to nearby heavy airstrikes during the 2024 Lebanon war, which lasted for 66 days. To note, our patient was only a few meters away from nearby airstrikes, during which she experienced the intense auditory stimulus of the explosion and significant acute emotional distress.

Even though causation cannot be established, the underlying mechanism of acute emotional stress as a precipitating factor is believed to involve dysregulation of the brain–heart axis, catecholamine surge, and coronary microvascular dysfunction, causing transient myocardial stunning [2, 8].

Notably, the effect of war on maternal health encompasses beyond direct physical injury. Women are disproportionately affected in conflict settings by indirect consequences, including displacement, disruption of healthcare systems, significant psychological stress, and reduced access to care [9]. Recent studies in perinatal populations exposed to violent conflicts show high rates of anxiety, depression, and post‐traumatic stress disorder (PTSD), with the latter potentially involved in adverse cardiovascular outcomes [10, 11].

In the postpartum, these stressors may be intensified by surgical stress, physiological changes, hormonal fluctuations, and sleep disturbances, establishing a vulnerable state where stress‐mediated cardiac impairment may occur [12]. Although evidence directly linking armed conflict to Takotsubo cardiomyopathy remains limited, the association seen in this case is biologically probable and consistent with the stress‐related pathophysiology of the condition.

Management of peripartum Takotsubo syndrome is mainly supportive and follows heart‐failure standards, including beta‐blockers, diuretics, and afterload reduction if needed [4].

This case emphasizes the importance of recognizing reverse Takotsubo syndrome in the differential of acute postpartum heart failure, especially in younger populations with regional wall‐motion abnormalities and speedy recovery.

Moreover, it further underscores the need to identify the broader health effects of violent conflicts, including possible stress‐mediated cardiovascular implications in vulnerable populations.

## Author Contributions

**Ali Khalil:** conceptualization, writing – review and editing, supervision. **Ghina Ghazeeri:** supervision, validation, writing – review and editing, conceptualization. **George Hatoum:** writing – original draft, resources. **Salwa Abouljoud:** writing – original draft, writing – review and editing. **Nathalie Chamseddine:** writing – original draft, writing – review and editing, resources. **Omar Alameddine:** writing – original draft, resources.

## Funding

The authors have nothing to report.

## Ethics Statement

Ethics approval is not required for case reports or case series deemed not to constitute research at our institution.

## Consent

Written informed consent was obtained from the patient for publication of this case report and any accompanying images.

## Data Availability

Data sharing not applicable to this article as no datasets were generated or analysed during the current study.
